# Hemorrhagic Diathesis

**Published:** 1875-07

**Authors:** S. B. Chase

**Affiliations:** Osage, Iowa.


### ARTICLE VI.

### *Hemmorrhagic Diathesis.*

BY S. B. CHASE, M. D., OF OSAGE, IOWA.

Reviewing some notes of the following case, I thought they might not be uninteresting to your readers.

On Tuesday, July 23d, 1872, I extracted the wisdom tooth from the left side of the lower jaw of A. M. Bryson, a lawyer of this city. Mr. B. is an American, about thirty years age, of full habit, weighing one hundred and sixty pounds, and perfectly healthy.

At the time I extracted the tooth, he informed me he came near bleeding to death from extraction of the tooth in front of it. The first molar had been previously drawn without special hemorrhage. At the time the second was extracted, having bled about three weeks, in defiance of the best medical aid at command, and until nearly exhausted, he stopped the hemorrhage by holding the finger firmly pressed into the cavity, about twelve hours, with the upper teeth shut hard upon it.

I had a vivid recollection of having treated him for epistaxis, in which I succeeded in checking the hemorrhage with full doses of tinct. ferri mur. internally, after having failed with other remedies.

I also remembered having barely saved the life of his little daughter, aged three years, in the winter of 1869 and and 1870, from profuse uterine hemorrhage. She had three successive attacks, at intervals of about four weeks each, ushered in with the usual accompaniments of puberty. This little girl had chicken-pox during the time, and the pustules filled with blood almost as soon as raised. In this case, as in the father's, the tinct. ferri mur. succeeded after other remedies failed.

Mr. B. informed me that he had a brother who had bled severely from the kidneys and bowels for more than a year, occasioned by jumping from a horse.

Although aware of these facts, and admitting the possible danger, I fearlessly extracted the tooth, not entertaining a doubt but I could readily stop the bleeding, should it occur.

The gum appeared healthy, and the tooth came easily; nor did ten drops of blood follow its extraction. After remaining in the office about half an hour, greatly rejoiced at the success, as he had suffered some time from the tooth, fearing to have it drawn, he left for home, free from pain or indications of bleeding.

After about one hour, without premonition, the blood burst forth with great violence. Continuing to bleed profusely, he returned to my office, as I had directed. I filled the cavity with ferri persulph. which at once checked the hemorrhage, and he returned home, more pleased, if possible than at first, both believing the trouble ended.

He went about his usual business that day, and slept well that night. Early next morning the blood again gushed forth. The iron was again used, with perfect success for about ten hours, when the bleeding again burst forth. The cavity was repeatedly plugged, and the iron continued, with slight variation, until Saturday, each period of success shortening, until plugging, compression, and astringents, in every form, appeared to lose control of the hemorrhage.

Every known remedy, both external and internal, was tried without avail. After having been checked a short time the blood would pour out as violently as at the first. It began to ooze from the gums as from a sponge, and by Tuesday evening in defiance of astringents and tonics, aided by the most nutritious diet, the face began to assume a cadaverous expression; the extremitios became cold, and the outlook was about as cheerless as one could well desire. The sufferer had not lain down for a week, and the indication that "tired nature" was about ready to yield the contest was painfully apparent, all the physicians in the place generously aiding in the watchful care, both day and night.

At this stage we used nutmeg, browned like coffee, the panacea of a soldier, and with most astonishing success. The bleeding stopped, as by magic, for about six hours, when out it burst again, as fresh as at first. We repeated this remedy some four or five times, at each time with shortening intervals, until its power was gone. A strong solution of collodion, with all the tannic acid it would hold succeeded for a number of hours once, but for once only.

A free use of kino in the cavity and about the gums, with my finger pressed as firmly upon it, for a number of hours, as the patient could bear, partially checked the hemorrhage: yet a combination of kino, catechu, and tannin appeared to increase the bleeding.

After Tuesday we so far controlled the hemorrhage, that on the whole we made blood faster than the sufferer lost it, thus passing the danger, though by no means relieving the the care or anxiety.

Here was a case in which the "sure cure" of "the oldest and wisest inhabitant" failed, "the vanity of earthly wisdom."

The actual cautery was not tried, as it had signally failed in his previous case. The most vigorous application of arg. nit., used at the earnest request of an able physician, increased the trouble. After thirteen days of doubtful struggle, we finally succeeded with the same remedy which had repeatedly failed us, a plug of persulphate of iron, yet not until the patient had bled at least eight quarts. In a medical journey of about twenty-five years I thought I had traveled over as hard spots as I would be likely to meet, yet can earnestly say, give me a repetition of any of them rather than anothse job like this.—*Med. & Surg. Reporter.*

